# Lysophosphatidic Acid Induces Aerobic Glycolysis, Lipogenesis, and Increased Amino Acid Uptake in BV-2 Microglia

**DOI:** 10.3390/ijms22041968

**Published:** 2021-02-17

**Authors:** Lisha Joshi, Ioanna Plastira, Eva Bernhart, Helga Reicher, Chintan N. Koyani, Tobias Madl, Corina Madreiter-Sokolowski, Zhanat Koshenov, Wolfgang F. Graier, Seth Hallström, Wolfgang Sattler

**Affiliations:** 1 Division of Molecular Biology and Biochemistry, Gottfried Schatz Research Center, Medical University of Graz, 8010 Graz, Austria; lisha.joshi@medunigraz.at (L.J.); ioanna.plastira@medunigraz.at (I.P.); eva.bernhart@medunigraz.at (E.B.); helga.reicher@medunigraz.at (H.R.); cnkoyani@yahoo.com (C.N.K.); tobias.madl@medunigraz.at (T.M.); corina.madreiter@medunigraz.at (C.M.-S.); zhanat.koshenov@medunigraz.at (Z.K.); wolfgang.graier@medunigraz.at (W.F.G.); 2 BioTechMed Graz, 8010 Graz, Austria; 3 Division of Physiological Chemistry, Otto Loewi Research Center, Medical University of Graz, 8010 Graz, Austria; seth.hallstroem@medunigraz.at

**Keywords:** ACC, aerobic glycolysis, Akt, fatty acids, Hif1α, mTOR

## Abstract

Lysophosphatidic acid (LPA) species are a family of bioactive lipids that transmit signals via six cognate G protein-coupled receptors, which are required for brain development and function of the nervous system. LPA affects the function of all cell types in the brain and can display beneficial or detrimental effects on microglia function. During earlier studies we reported that LPA treatment of microglia induces polarization towards a neurotoxic phenotype. In the present study we investigated whether these alterations are accompanied by the induction of a specific immunometabolic phenotype in LPA-treated BV-2 microglia. In response to LPA (1 µM) we observed slightly decreased mitochondrial respiration, increased lactate secretion and reduced ATP/ADP ratios indicating a switch towards aerobic glycolysis. Pathway analyses demonstrated induction of the Akt-mTOR-Hif1α axis under normoxic conditions. LPA treatment resulted in dephosphorylation of AMP-activated kinase, de-repression of acetyl-CoA-carboxylase and increased fatty acid content in the phospholipid and triacylglycerol fraction of BV-2 microglia lipid extracts, indicating de novo lipogenesis. LPA led to increased intracellular amino acid content at one or more time points. Finally, we observed LPA-dependent generation of reactive oxygen species (ROS), phosphorylation of nuclear factor erythroid 2–related factor 2 (Nrf2), upregulated protein expression of the Nrf2 target regulatory subunit of glutamate-cysteine ligase and increased glutathione synthesis. Our observations suggest that LPA, as a bioactive lipid, induces subtle alterations of the immunometabolic program in BV-2 microglia.

## 1. Introduction

Microglia, the immune competent cells of the brain arise from the mesoderm lineage and colonize the neuroepithelium by embryonic day 9.5 [1]. Microglia represent a self-renewing population of cells and play fundamental roles during surveillance of CNS homeostasis [2]. Neurodegenerative disorders that are characterized by an inflammatory response [3] are accompanied by activation of the innate immune cells of the CNS. During the inflammatory response, microglia can adopt different polarization states ranging from a pro-inflammatory to an immunosuppressive/anti-inflammatory phenotype [4]. Depending on the outcome of the polarization program, microglia can aggravate disease or support neuronal survival [5,6]. Combination of sophisticated analytical methods, such as RNAseq, transcriptomic, and proteomic analysis of microglia at a single-cell level revealed unique phenotypic signatures in physiological and neurodegenerative settings [7,8,9]. These studies demonstrated that microglia are highly dynamic cells even in their “resting” state [10]. Depending on the environmental input via a specific set of receptors termed as the microglia sensome [11] these cells are primed to perform different functions including phagocytosis, inflammasome activation, and/or cyto-/chemokine, reactive oxygen- and nitrogen species release [12,13]. These responses are regulated through complex transcriptional and functional changes that determine polarization toward a neurotoxic or neuroprotective phenotype [14]. The pro-inflammatory immune response driven by microglia is considered a key contributor to the pathogenesis of neurodegenerative diseases [15].

The transient metabolic profile of immune cells has profound implications on the induction of pro- or anti-inflammatory phenotypes [16,17]. Within the field of immunometabolism several aspects of metabolic pathway utilization/adaption and the resulting immune cell phenotype in the periphery are clarified, however, this field is just emerging in microglia biology [16,18]. Transcriptomic studies indicate that microglia express transporters for glucose, glutamine, and fatty acids (FA) and like macrophages, microglia display metabolic flexibility and are able to rewire energy fuel utilization [19]. In the absence of glucose microglia are able to consume glutamine as an alternative nutrient source, while the contribution of β-oxidation to cellular energy homeostasis generation is not entirely clear [16,20]. To get an indication about basic metabolic pathway utilization of microglia some of these studies were performed in “unstimulated” cells resembling microglia in their surveillance state [21,22]. In response to extracellular signals, e.g., lipopolysaccharide (LPS), immune cells are able to mount an immune response of different strength and duration. It is becoming increasingly clear that the type, strength, and duration of this response (i.e., the polarization phenotype) is tightly controlled by metabolic pathway adaptation and rewiring [23,24]. Macrophages that are stimulated by LPS increase their reliance on glycolysis as a necessary adaptation during proinflammatory activation [25] and similar observations were reported for microglia [26]. Under aglycemic conditions it was demonstrated that microglia are able to switch from glycolysis to glutaminolysis in an mTOR-dependent manner indicating metabolic plasticity [20].

During earlier and ongoing studies our group could show that lysophosphatidic acid (LPA) represents an extracellular signal that induces polarization of a microglia cell line (BV-2) and primary murine microglia towards a neuroinflammatory phenotype in vitro [27,28]. In the CNS this bioactive lipid is detected in embryonic brain, choroid plexus, meninges, neural tube, blood vessels, spinal cord, and cerebrospinal fluid (CSF) at nanomolar to micromolar concentrations [29]. LPA signal transduction is facilitated by specific G protein-coupled LPA receptors (termed LPAR1-6) that mediate the diverse effects [30]. Under physiological conditions LPA-mediated signaling is essential for normal neurogenesis and function of the CNS. In response to injury LPA concentrations increase in brain and CSF [31,32,33,34,35]. Aberrant LPA signaling contributes to multiple disease states, including neuropathic pain, neurodegenerative, neurodevelopmental, and neuropsychiatric disorders [30]. In microglia many inflammatory effects of LPA are transmitted via LPAR5 [6,28,36], which was identified as a member of the microglia sensome [11].

During an earlier proteome study in a human microglia cell line, we could show that lysophosphatidic acid (LPA) activation induces prominent changes in the expression of proteins that regulate cell motility and/or cytoskeletal dynamics [37]. In the context of immunometabolism it is important that LPA treatment of this microglia cell line potently induced expression of several glycolytic enzymes, an observation accompanied by increased cell locomotion [37]. These findings indicate that LPA represents an extrinsic factor that is able to induce metabolic programming in microglia. Murine BV-2 cells express LPAR2, -3, -5, and -6 mRNA (with LPAR5 and 6 being most abundant) and receptor expression is not affected by exogenously added LPA [27]. In contrast, LPA induced expression of M1 markers and the secretion of pro-inflammatory cyto-/chemokines and increased NO and ROS production by BV-2 cells, indicating polarization towards a neurotoxic phenotype [27]. BV-2 cells express autotaxin and secrete LPA in response to lipopolysaccharide (LPS) treatment [38]. These findings suggest that BV-2 cells represent a suitable in vitro model to study phenotypic changes that are induced by LPA.

In the present study we report that LPA shifts BV-2 cell metabolism towards increased lactate production reminiscent of what was reported for M1 macrophages or amyloid-β (Aβ)-treated primary microglia. LPA treatment decreased mitochondrial function and the ATP/ADP ratio, activated the protein kinase B (AKT)—mammalian target of rapamycin (mTOR)—hypoxia-inducible factor-1α (HIF1α) pathway, and induced de-phosphorylation (de-repression) of acetyl-CoA carboxylase (ACC), observations accompanied by increased lipid synthesis and intracellular amino acid concentrations. Finally, we found that LPA triggered ROS production, nuclear factor erythroid 2–related factor 2 (Nrf2) phosphorylation, and enhanced expression of the downstream target glutamate-cysteine ligase modifier subunit (GCLm). These signaling events were accompanied by transiently increased glutathione (GSH) content. Our findings in BV-2 cells suggest that LPA as a proinflammatory signal is also able to induce metabolic reprogramming in microglia.

## 2. Results

### 2.1. LPA Impacts Energy Metabolism of BV-2 Cells

**Figure 1:**
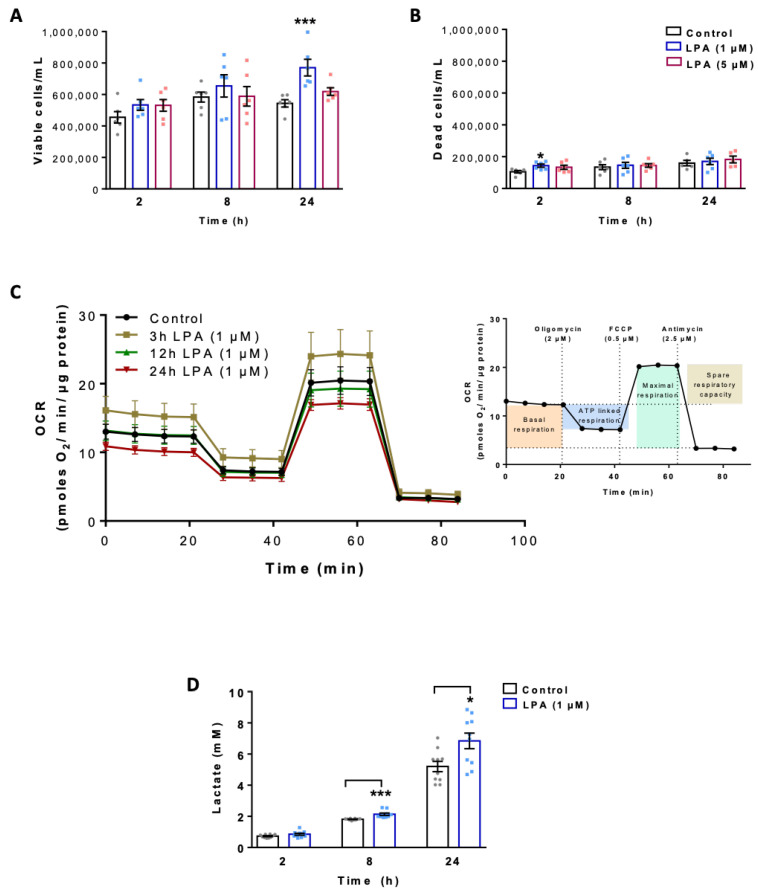
Effect of lysophosphatidic acid (LPA) on cell viability, mitochondrial respiration and lactate content of BV-2 microglia. The numbers of (**A**) viable and (**B**) dead cells were determined by Guava ViaCount analysis in the absence and presence of LPA. (**C**) Oxygen consumption rate (OCR) in the absence and presence of 1µM LPA for the indicated times was detected using the XF Cell Mito Stress Test. Cells were treated with 2 μM oligomycin, 0.5 μM FCCP, and 2.5 μM antimycin A in XF assay medium to assess fundamental parameters of mitochondrial function (inset). (**D**) Lactate content in the supernatants of LPA (1 µM) treated cells was measured by EnzyChrom™ Glycolysis Assay Kit and compared to their appropriate controls. Bar graph represents mean values ± SEM of 3 independent experiments; (* *p* < 0.05, *** *p* < 0.001 compared to control; one-way ANOVA with Bonferroni correction).

In a first set of experiments, we performed proliferation and viability studies in untreated and LPA-treated (1 and 5 µM) BV-2 cells using the ViaCount system. Viable cell numbers were significantly increased by 1 µM LPA at 24 h (Figure 1A). The number of dead cells (roughly 20% of total cell counts due to prolonged exposure to serum free conditions) was unaffected (except at 2 h, 1 µM) by LPA (Figure 1B).

To investigate the mitochondrial response of BV-2 cells toward LPA, we analyzed the oxygen consumption rate (OCR) using the real time Seahorse Extracellular Flux Analyzer mitochondrial stress test (Figure 1C). This protocol uses successive application of oligomycin (an inhibitor of the ATP synthase that reveals ATP-linked respiration), FCCP (a protonophore that drives maximal respiration), and rotenone/antimycin A (inhibitors of complex I and III blocking electron chain transport (ETC) activity) to quantitate key parameters of mitochondrial function (inset in Figure 1C). Cells were plated in Seahorse XF cell culture microplates in the absence or presence of LPA (1µM) for the indicated times and OCR was measured. These experiments revealed that acute LPA exposure for 3h led to a transient increase in OCR, while at longer incubation times basal and maximal OCR showed a (non-significant) trend for a decrease (Figure S1A,B). A similar pattern was observed for mitochondrial ATP production (Figure S1C) and the spare respiratory capacity (Figure S1D). Although these findings suggest decreased mitochondrial function of BV-2 cells in response to LPA (at incubation times > 12 h) the data did not reach statistical significance. However, diminished MTT reduction in LPA-treated cells (1 µM) is of support for decreased mitochondrial activity (Figure S1E). In a separate set of experiments, we examined the levels of secreted lactic acid, the major end-product of aerobic glycolysis. These analyses revealed that the media concentrations of lactate were significantly higher (6.8 vs. 5.2 mM) in BV-2 cells exposed to LPA for 8 and 24 h, indicating a shift toward aerobic glycolysis (Figure 1D).

**Figure 2:**
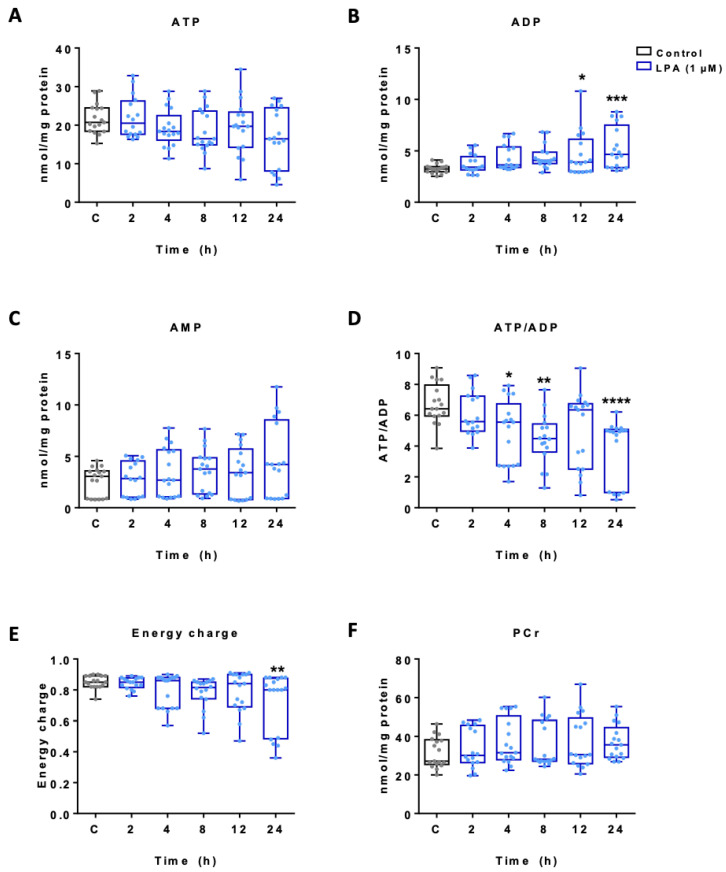
Effect of LPA on adenine nucleotide and phosphocreatine content in BV-2 microglia. Total cellular adenine nucleotide and phosphocreatine (PCr) content of BV-2 microglia incubated in the absence (c) and presence of 1 µM LPA was quantified using HPLC. Graphs represent the concentration of (**A**) ATP, (**B**) ADP, (**C**) AMP, (**D**) ATP/ADP ratio, (**E**) the energy charge (EC) calculated as EC = ([ATP + 1/2ADP]/[ATP + ADP + AMP]) and (**F**) phosphocreatine concentration. Results are presented as mean values ± SEM of 3 independent experiments. (* *p* < 0.05, ** *p* < 0.01, *** *p* < 0.001, **** *p* < 0.0001 compared to control; one-way ANOVA with Bonferroni correction).

To get an indication about total cellular adenine nucleotide and phosphocreatine (PCr) concentrations HPLC analyses were performed. The data presented in Figure 2A show reduced ATP content (statistically not significant) with a concomitant significant increase in ADP at 12 and 24 h (Figure 2B) while the changes in AMP concentrations were not significant (Figure 2C). Consequently, LPA treatment of BV-2 cells resulted in a decreased ATP/ADP ratio (Figure 2D) and a decline in energy charge (Figure 2E). PCr concentrations remained unchanged (Figure 2F).

### 2.2. LPA Activates Akt and mTOR and Induces HIF1α Expression

**Figure 3:**
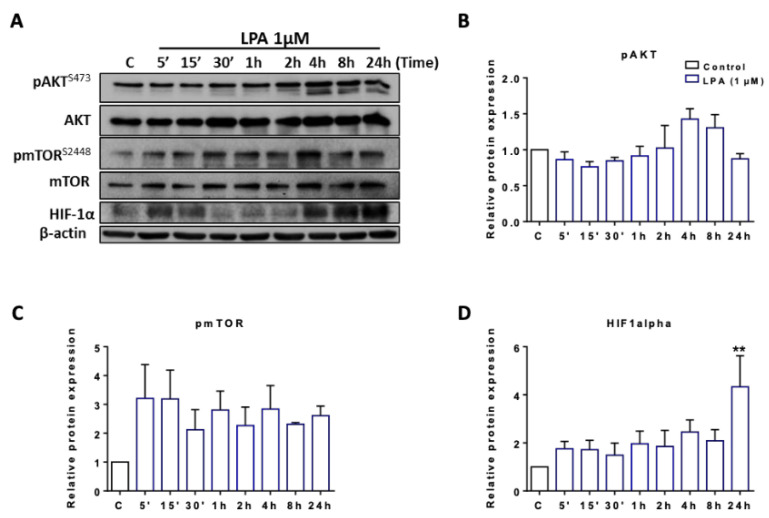
Immunoblot analysis of proteins involved in glycolysis upon treatment with LPA. (**A**) Serum-starved BV-2 cells were incubated in the absence (c) and presence of 1 µM LPA for the indicated time points. Expression levels of energy/oxygen sensing proteins were examined by immunoblotting. One representative blot out of three is shown. β-actin was used as loading control. Bar graphs represent densitometric analyses of immunoreactive bands of (**B**) phosphorylated AKT (pAKT), (**C**) phospho mTOR (pmTOR), and (**D**) HIF1α relative to β-actin. Results are presented as mean values ± SEM of 3 independent experiments. (** *p* < 0.01 compared to control; one-way ANOVA with Bonferroni correction).

We have previously shown that transduction of LPA signals via LPAR5 leads to activation of PKB/Akt [28]. Akt transmits extracellular signals and can increase mTOR phosphorylation, part of a mechanism sensing the energy status of a cell. Phosphorylated mTOR increases expression of HIF1α, the master regulator of glycolysis [39]. Therefore, we analyzed intracellular signaling pathways that converge on glycolysis by Western blot analyses. These experiments revealed LPA-induced phosphorylation of Akt and mTOR at incubation times >2 h without affecting total Akt and mTOR levels. In addition, LPA induced HIF1α expression with elevated levels maintained up to 24 h under normoxic conditions (Figure 3A; data of one representative experiment). Densitometric evaluations of band intensity from three independent biological replicates are shown in the bar graphs in Figure 3B–D. These findings suggest that LPA activates a signaling axis in BV-2 cells that induces aerobic glycolysis (the “Warburg effect”).

Since HIF1α was shown to regulate transcription of glucose transporters (GLUT) in microglia we performed qPCR analyses of LPA stimulated BV-2 cells. These data showed a trend for transient, LPA-mediated upregulation of GLUT1 (highest expression of all GLUTs in BV-2; [40]), GLUT3, GLUT4, GLUT5, and HK2 gene expression. However, none of the changes reached statistical significance (Figure S2).

### 2.3. LPA Induces Lipogenesis in BV-2 Cells

**Figure 4:**
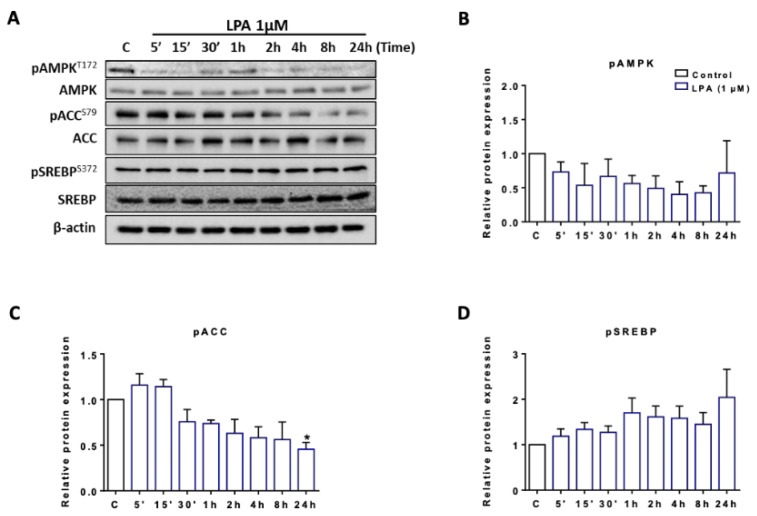
Phosphorylation status of AMP-activated protein kinase (AMPK), acetyl-CoA carboxylase (ACC), and sterol regulatory element binding protein (SREBP) in LPA treated BV-2 cells. (**A**) BV-2 cells were serum-starved overnight and incubated in the absence (‘c’) and presence of 1 µM LPA for the indicated times. Phosphorylation states of proteins were detected using Western blot analysis, β-actin served as loading control. One representative blot out of three is shown. Densitometric analyses of immunoreactive bands of (**B**) phospho AMPK (pAMPK), (**C**) phospho ACC (pACC), and (**D**) phospho SREBP (pSREBP) relative to β-actin are shown. Results are presented as mean values ± SEM of 3 independent experiments. (* *p* < 0.05, compared to control; one-way ANOVA with Bonferroni correction).

Microglia engage actively in lipid and lipoprotein metabolism [41] since pro- or anti-inflammatory stimuli induce constant morphological transitions and (micro)vesicle-mediated secretion of cytokines [12]. It is reasonable to assume that these processes govern (membrane) lipid remodeling pathways [42,43]. Therefore, we investigated signaling pathways and the FA composition of major lipid subclasses in naive and LPA-stimulated BV-2 cells to get basic insight in such processes. We first studied the phosphorylation status of key regulators of fatty acid turnover. AMP-activated protein kinase (AMPK) is a “metabolic master switch” and a central regulator of acetyl-CoA carboxylase 1 (ACC1) activity, a key enzyme involved in fatty acid synthesis. AMPK-dependent phosphorylation of ACC1 at S^79^ inhibits malonyl-CoA formation and thereby FA synthesis/elongation. Analysis of AMPK phosphorylation (AMPK^T172^) by Western blotting revealed a time dependent reduction of S^172^ phosphorylation in response to LPA with total immunoreactive AMPK remaining unchanged (Figure 4A). This was accompanied by ACC1^S79^ dephosphorylation (at time points > 30 min) indicating ACC1 de-repression. S^372^ phosphorylation of the sterol regulatory element binding protein (SREBP)-1c precursor was not significantly changed (Figure 4A). Densitometric evaluations of band intensity from three independent biological replicates are shown in the bar graphs in Figure 4B–D.

**Figure 5:**
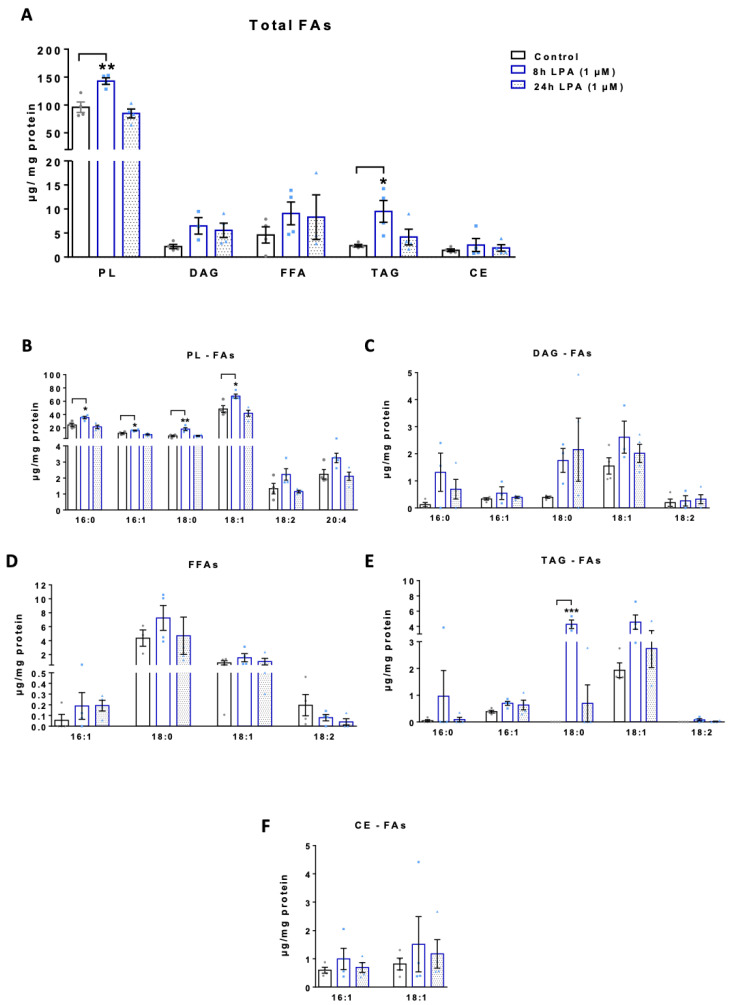
Quantitation of fatty acid species in lipid subclasses of LPA treated BV-2 cells. Serum starved BV-2 cells were incubated in the absence and presence of 1 µM LPA for the indicated times. (**A**) Quantitation of total FAs present in the indicated lipid species (PL, phospholipids; DG, diacylglycerols; FFA, free fatty acids; TAG, triacylglycerols; CE, cholesterylesters). Individual FA content in each lipid class: (**B**) Phospholipids, (**C**) diacylglycerols, (**D**) free fatty acids, (**E**) triacylglycerols, and (**F**) cholesterylesters. Data represent mean ± SEM of four independent experiments; * *p* < 0.05, ** *p* < 0.01, *** *p* < 0.001 compared to control; one-way ANOVA with Bonferroni correction).

We then moved on to quantitate the FA content in major lipid subclasses (phospholipids, PL; free fatty acids, FFA; diacylglycerols, DAG; triacylglycerols, TAG; and cholesterylesters, CE) isolated from untreated and LPA-treated (8 and 24 h) BV-2 cells. Under basal conditions highest concentrations were detected for the PL fraction (96 µg FA/mg cell protein), while the other lipid subclasses were less abundant (Figure 5A). Of note, the total FA content in BV-2 cells under basal conditions is in good agreement with published data [44]. The total FA content in the PL and TAG fraction was significantly increased 8h post LPA, while the FFA, DAG, and CE fraction only showed a tendency for an increase (Figure 5A). Within PLs 16:0, 16:1, 18:0, and 18:1 was significantly increased (Figure 5B). In the DAG fraction 16:0, 18:0, and 18:1 were the predominating representatives (Figure 5C) while in the FFA fraction 18:0 was most abundant (Figure 5D). In the TAG family 18:0 and 18:1 was detected in the highest concentrations (Figure 5E) and in the CE subclass only CE16:1 and CE18:1 was detectable (Figure 5F) under the experimental conditions applied here.

### 2.4. LPA Increases Amino Acid Concentrations in BV-2 Cells

**Figure 6:**
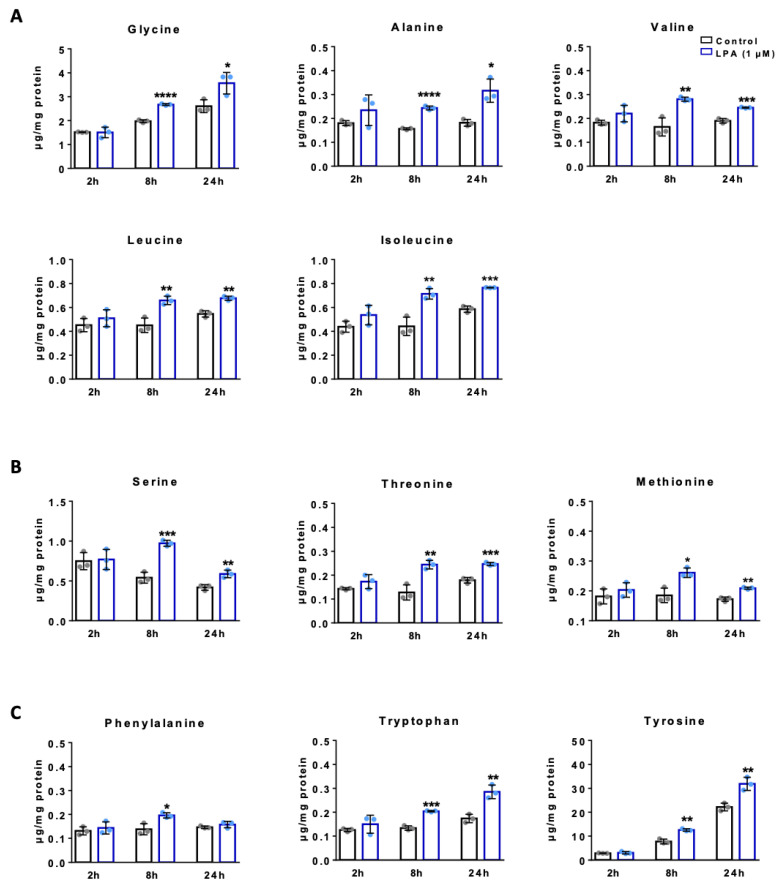

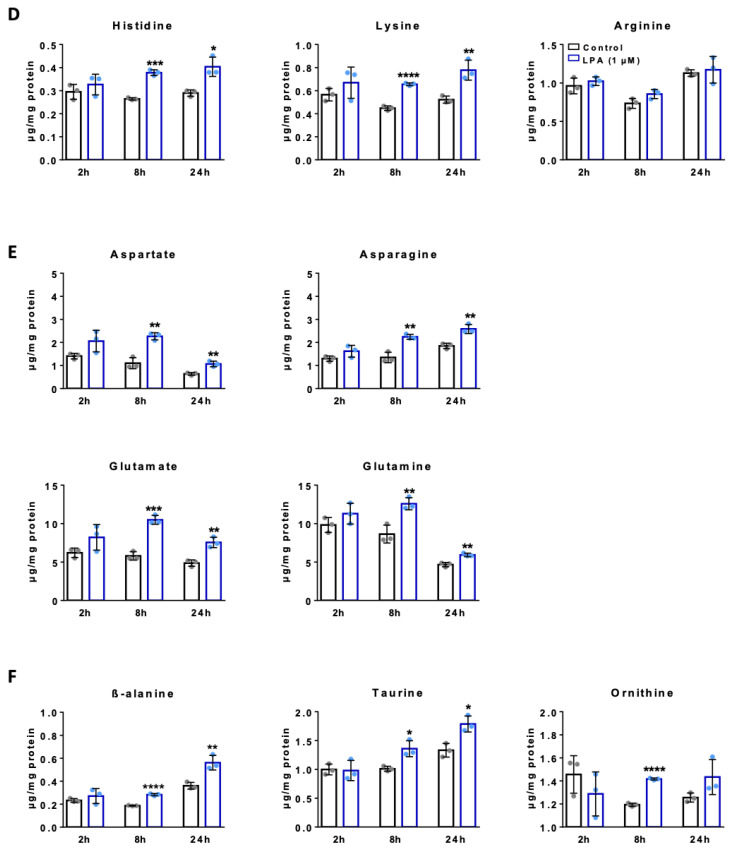
Amino acid analysis of LPA treated BV-2 cells. Serum-starved BV-2 cells were incubated in the absence (control) and presence of LPA (1 µM) for the indicated times. HPLC analysis of OPA derivatized amino acids (AAs) was performed and amino acid concentrations were determined by area comparison with external calibration curves and subsequent normalization to the cellular protein content. Bar graphs indicate mean ± SEM of 3 independent experiments. (* *p* < 0.05, ** *p* < 0.01, *** *p* < 0.001, **** *p* < 0.0001 compared to controls; *Student’s* *t*-test). The amino acids are grouped according to their chemical properties in: (**A**) Aliphatic AAs, (**B**) OH- and S-containing AAs, (**C**) aromatic AAs, (**D**) basic AAs, (**E**) acidic AAs, and (**F**) non-proteogenic AAs.

LPA-mediated microglia activation is tightly coupled to protein synthesis, which is necessary for cytokine production, and it is conceivable this results in a net increase of amino acid (AA) uptake. In addition, AAs activate mTOR signaling and are funneled into metabolic intermediates that participate in a variety of metabolic processes including fatty acid synthesis [45]. Therefore, we determined time dependent turnover of free amino acids in BV-2 cells. Amino acids were separated as OPA derivatives by HPLC and quantitation was performed with fluorescence detection and peak area comparison with external standard mixtures. Under basal conditions Glu and Gln with 6 and 10 µg/mg cell protein were the most abundant amino acids, followed by Asp, Asn, Gly, and Tyr (Figure 6). The aliphatic AAs Gly, Ala, Val, Leu, and Ile increased in response to LPA treatment over the entire time course (Figure 6A) and the same is true for the second group (OH- and S-containing; Ser, Thr, and Met; Figure 6B). Also, the aromatic amino acids Phe, Tyr and Trp increased in LPA-treated cells (Figure 6C). Within this class the pronounced time-dependent accumulation of Tyr in untreated and treated cells (>10-fold) is remarkable. Within the group of basic AAs His and Lys concentrations were elevated in treated cells, while Arg was almost unaffected (Figure 6D). The acidic AAs represented the quantitatively most abundant group and Asp, Asn, Glu, and Glu concentrations were significantly augmented in response to LPA (Figure 6E). In the last group of non-proteinogenic AAs β-Ala, Tau, and Orn were also increased by LPA (Figure 6F). The cyclic AA Pro is not amenable to OPA derivatization and Cys gives only a very low fluorescence response, therefore these AA are not included in the data sets below.

### 2.5. LPA Activates the Nrf2-Mediated Antioxidant Response in BV-2 Cells

**Figure 7:**
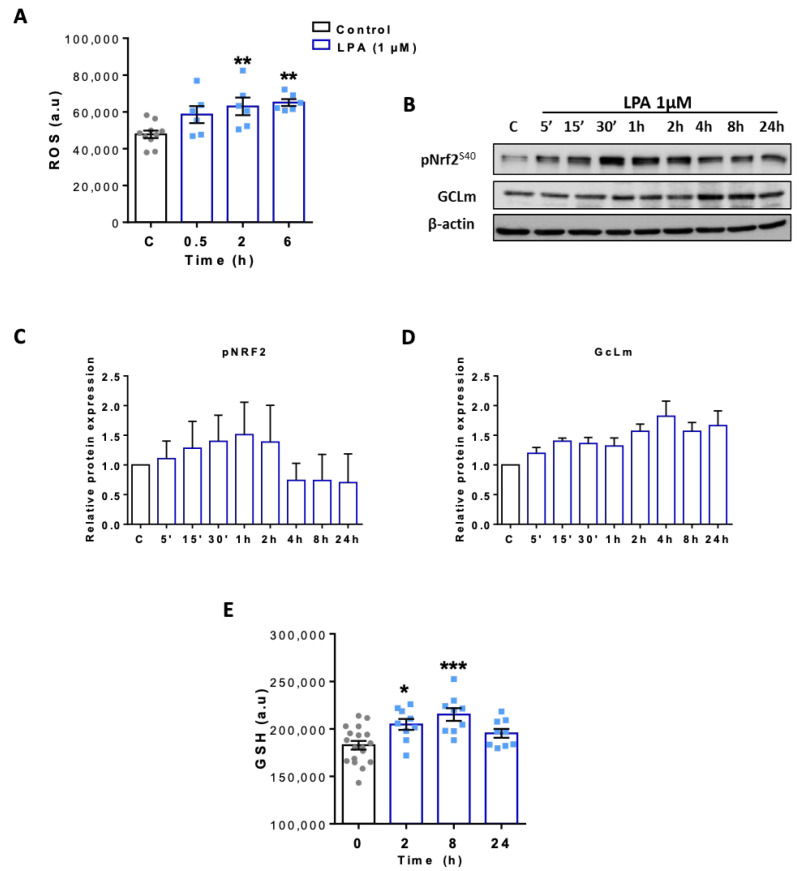
Characterization of the antioxidant response in LPA treated BV-2 cells. (**A**) Reactive oxygen species (ROS) of control and LPA (1 µM) treated cells was quantified using the ROS-ID^®^ Total ROS Detection kit (a.u. arbitrary units). (**B**) Immunoblot analysis of phospho Nrf2 (Ser40) and GCLm upon treatment with LPA. One representative blot out of three is shown. β-actin was used as loading control. (**C**,**D**) Densitometric analysis relative to β-actin from three independent immunoblot experiments are presented. (**E**) Glutathione (GSH) concentration of LPA (1µM) treated cells was quantified with GSH-Glo Assay kit and compared with controls in three independent experiments. Results are presented as mean values ± SEM (* *p* < 0.05, ** *p* < 0.01, *** *p* < 0.001, compared to control; one-way ANOVA with Bonferroni correction).

As shown earlier LPA treatment induces ROS generation in BV-2 cells (Figure 7A). To clarify whether ROS generation leads to an antioxidant response, activation of Nrf2 was studied by Western blotting experiments. As shown in Figure 7B,C, LPA treatment induces phosphorylation of Nrf2 at S^40^, a critical signaling event leading to the ARE-mediated cellular antioxidant response [46]. In line, we observed increased expression of glutamate-cysteine ligase light chain (GCLm; Figure 7B,D), the modifier subunit of GCL which is the rate-limiting enzyme for GSH synthesis and a transcriptional Nrf2 target. This was reflected on product level since GSH concentrations were significantly increased 2 and 8h post LPA treatment (Figure 7E).

Using LPA at 5 µM resulted in qualitatively comparable but quantitatively less pronounced effects: We observed a transient increase (at 3 h) of mitochondrial function in Seahorse experiments (Figure S3A–E), a decrease in MTT reduction (Figure S3F), significantly increased lactate production (Figure S3G), less pronounced alterations in nucleotide content (Figure S4A–E), and unchanged PCr concentrations (Figure S4F). Also, the induction of the Akt/mTOR/Hif1a axis was less pronounced but a significant induction of GLUT1 and HK2 mRNA was observed (Figure S5A–F). Changes in AMPK and ACC phosphorylation were lower and (despite a trend for increase) no statistically significant changes in lipid content were observed (Figure S6A–G). ROS levels in untreated and treated cells were similar (Figure S7A), while pNrf2, GCLm, and GSH levels were elevated (Figure S7B,C). No differences in amino acid concentrations were observed between untreated and treated (5 µM) cells (data not shown).

## 3. Discussion

Recent studies implicate the ATX-LPA-LPAR axis as a central signaling hub that induces metabolic reprograming. This was shown in mice where heterozygous ATX (gene name Enpp2) knockout protected from diet-induced obesity and insulin-resistance and restored mitochondrial function in skeletal muscle [47], or in adipocyte-specific Enpp2^-/-^mice that, on a high-fat diet, showed smaller body weight gain and less insulin resistance than control mice [48]. In ovarian cancer models, LPA induces a shift towards glycolysis [49], upregulates hexokinase 2 [50], and induces metabolic reprograming via a pseudo-hypoxic response that is HIF1α-mediated [51]. In the brain, LPA mediates diverse biological actions on different cell types including neurons, astrocytes, oligodendrocytes, and microglia [52]. In microglia LPA can have protective [38,53] or detrimental effects [6,27], the outcome presumably depending on the cellular pre-activation state. BV2 microglia that were used throughout the present study are a well-characterized and widely used in vitro system for microglia studies [54]. BV-2 cells express all the functional elements necessary to study ATX/LPA/LPAR signaling events in microglia, have, however, also clear limitations since they represent a murine, v-raf/v-myc carrying J2 retrovirus transformed cell line [55,56]. Earlier studies addressing the role of LPA in microglia polarization revealed that, on a qualitative basis, BV-2 and primary cell responses were comparable but quantitatively less pronounced in BV-2 cells [27]. This quantitative difference was also reported for LPS-stimulated BV-2 and primary microglia [57], while an inflammatory response towards Aβ42 was nearly absent in BV-2 but highly induced in primary microglia [58]. Thus, it is clear that BV-2 cells are only a surrogate model that cannot fully replace primary microglia.

**Figure 8:**
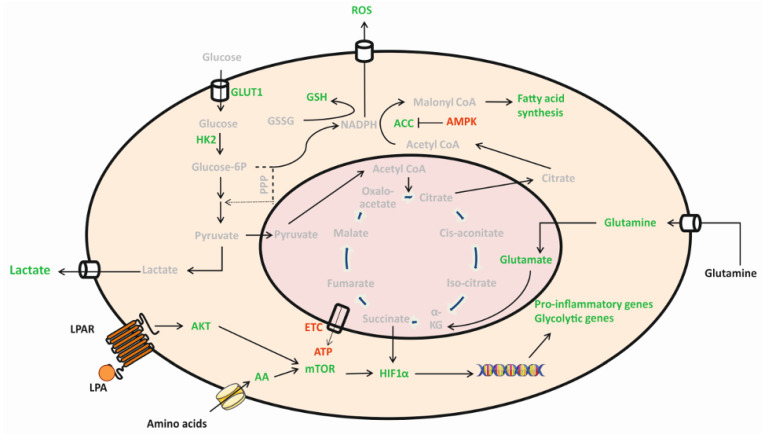
Key metabolic pathways alterations in LPA-activated BV-2 microglia. Graphical summary of findings obtained during this study. Stimulation of microglia with LPA reprograms metabolism towards aerobic glycolysis for generation of ATP via the Akt/mTOR/HIF1α axis. As a consequence, lactate secretion increases. The activated BV-2 cells switch mitochondrial metabolism to support FA synthesis. Increased AA uptake can further fuel this metabolic program and sustain proinflammatory cytokine synthesis. In response to ROS generation the antioxidant response is initiated and results in higher levels of intracellular GSH to prevent damage of endogenous macromolecules. Red = decreased, green = increased. (Intermediates not experimentally addressed during the present study are shown in grey).

LPA treatment of BV-2 and primary murine microglia induces activation of pro-inflammatory signaling cascades, secretion of chemo-/cytokines, induction of ROS production and M1 plasma membrane marker expression indicating polarization towards a neurotoxic phenotype [6,27,28]. To investigate whether these alterations are accompanied by the induction of a specific immunometabolic phenotype we investigated specific aspects of energy metabolism in LPA-treated BV-2 microglia. Our major findings can be summarized as follows: LPA was without inadvertent effects on cell viability, increased proliferation slightly, induced a (non-significant) decrease in mitochondrial respiration, increased extracellular lactate concentration, decreased the cellular ATP content and activated the Akt-mTOR-Hif1α axis under normoxic conditions. While some of the events relevant for glycolysis were statistically not significant, alterations in lipid metabolism (de-repression of ACC and significantly increased FA concentrations present in the PL and TAG fraction) were more clear-cut. Also, the free amino acid content of BV-2 cells was increased in response to LPA. Most pronounced increases were observed for Asp, Asn, Glu, and Ser (≥2-fold 8h post LPA application). Finally, we show LPA-dependent ROS generation, phosphorylation of Nrf2, upregulation of the antioxidant response element (ARE) target GCLm and increased GSH synthesis. Of note, LPA at 5 µM resulted in qualitatively comparable but quantitatively less pronounced responses as compared to 1 µM LPA. Whether this is caused by LPA receptor desensitization (discussed below) is currently unclear. A graphical summary of the findings obtained during the present study with 1 µM LPA is presented in Figure 8.

The highly dynamic nature of microglia that is associated with a constant remodeling of the cytoskeleton indicates an energy-demanding metabolic phenotype [59]. On basis of transcriptome studies, it was suggested that microglia express all enzymes necessary to cover the major metabolic pathways [21,60]. Indeed, the bioenergetic microglia machinery is able to utilize glucose, glutamine, pyruvate, lactate, ketone bodies, or fatty acids as energy substrates [16,59,61]. Recent in vivo studies revealed metabolic flexibility of microglia that, under glucose deprived conditions, can switch to glutaminolysis in an mTOR-dependent manner [16,20]. All of these pathways converge on oxidative metabolism in mitochondria under physiological conditions. However, in neurodegeneration mitochondrial dysfunction plays a major role in disease pathogenesis [62]. This is accompanied by mitochondrial hypometabolism and aberrant cellular redox control, as well as a decrease of ATP levels in brains of experimental AD mouse models [63]. In line amyloid-β acutely triggers inflammatory microglia activation accompanied by a switch from Oxphos towards aerobic glycolysis in an Akt-mTOR-Hif1α -mediated pathway [64].

This is reminiscent of what we observed during the present study in LPA-activated BV-2 microglia (Figure 1, Figure 2, Figure 3 and Figure 4). During an earlier proteome study in a human microglia cell line, we could show that LPA treatment induced upregulated expression of several enzymes primarily active in the triose branch of glycolysis [37]. Here we show that this pathway involves Akt, mTOR, and Hif1α in a murine microglia cell line. In LPA-stimulated primary murine microglia Akt activation occurs via protein kinase D2 [28] and this cascade presumably leads to downstream mTOR/HIF1α activation [65]. In ovarian cancer cells it was demonstrated that LPA induces Hif1α activation via a pseudohypoxic response that leads to metabolic reprogramming towards aerobic glycolysis [51], while in colon cancer cells Hif1α induction is subject to reciprocal regulation by KLF5 and p53 [66]. In primary murine microglia it was shown that Hif1α activation induced upregulation of GLUT1 [67].

Interestingly, when BV-2 cells were incubated in the presence of 5 µM LPA the response was quantitatively less pronounced. These findings are reminiscent of receptor desensitization, a modulatory response of G protein-coupled receptor activation resulting from prolonged exposure to high agonist concentrations. Although not experimentally tested during the present study, reports describing (homologous) desensitization of LPA receptor signaling in Xenopus oocytes [68], fibroblasts [69], and hepatic epithelial cells would be in line with such an effect [70]. Desensitization would also be compatible with the bell-shaped concentration-dependent response of LPA-induced chemokinesis and chemotaxis of BV-2 cells: In these experiments both parameters reached a maximum at 1 µM and returned to baseline at 2 (chemotaxis) and 5 (chemokinesis) µM [28].

As a surveillance cell type microglia move their ramifications constantly indicating a high degree of membrane flexibility [10]. Since these movements are also associated with alterations of the membrane curvatures it is reasonable to assume that the phospholipid synthesis/degradation/remodeling machinery of this cell type must be able to respond to extracellular cues. Due to the specific tasks taken by different cell types in the brain it is conceivable that every cell type is characterized by a specific lipidome with microglia being enriched in sphingolipids and (ether) phospholipids [71]. During the present study we found that AMPK phosphorylation time dependently decreased and was accompanied by dephosphorylation (indicating de-repression) of ACC in response to LPA (Figure 5). This is reminiscent of what was reported in a murine endotoxemia model where LPS dose-dependently induced dephosphorylation of AMPK and its target protein ACC, an event accompanied by activation (phosphorylation at T^172^) of mTOR in lungs of LPS injected animals [72]. Dephosphorylation of ACC would then allow for synthesis of malonyl-CoA that fuels into de novo FA synthesis (Figure 6). This likely provides FA flux into increased PL, DAG/TAG, and CE synthesis as observed here in response to LPA (Figure 6A–E). All of these lipid families were recently identified in lipid droplets (LD) formed in vivo in aged microglia or in response to LPS treatment of young mice or BV-2 cells [73]. Of note Marschallinger and colleagues demonstrated that LD formation induces a proinflammatory (increased NO, ROS, and cytokine synthesis) microglia phenotype that is dysfunctional in terms of phagocytic capacity [73]. Microglia-located LD formation was also observed in a mouse model of Leigh Syndrome (deletion of Ndufs4, a mitochondrial Complex I subunit; [74]), ageing brain [75], and in LPS-treated N9-treated microglia [76].

We observed increased intracellular AA concentrations in LPA-treated BV-2 cells (Figure 7) and this might be a pathway replenishing substrate for LPA-induced cyto-/chemokine synthesis [6]. This observation is also of functional importance since mTOR signaling is activated by AA, a signaling event determining the fate of amino acid, glucose, nucleotide, and lipid metabolism [77]. mTOR is part of two functionally distinct complexes (mTORC1 and mTORC2) and within this scenario PI3K and subsequent Akt activation (as observed here) results in repression of TSC that leads to initial mTORC1 activation [65]. Final, full activation of mTORC1 requires import of amino acids in the cytoplasm. Among the AA that mediate full activation of mTORC1 are Leu, Gln, Glu, and Arg [65].

Gln and Glu were quantitatively the most abundant AA in naïve BV-2 cells and the intracellular concentrations were further increased by LPA (Figure 7E; >10µg/mg cell protein). As mentioned above microglia can use Gln as anaplerotic energy substrate in a process known as glutaminolysis. The first step in this pathway is catalyzed by one of the two isoforms of glutaminase (GLS1, GLS2) converting Gln to Glu. Brose and colleagues demonstrated that BV-2 cells are able to incorporate carbon from [^14^C]-labeled Gln and Glu into newly synthesized cellular lipids indicating that reductive carboxylation of Glu-derived α-ketoglutarate (αKG) is operative in mitochondria of this microglia cell line [78]. Subsequent export of citrate to the cytosol and hydrolysis by ATP-dependent citrate lyase yields AcCoA that serves as substrate for ACC and FASN. Catabolism of a second class of AA, namely the branched chain AA Val, Leu, and Ile can also fuel fatty acid synthesis and lipogenesis in adipocytes [45]. Thus, increased AA uptake as observed here can provide AA substrates for de novo cyto-/chemokine synthesis, contribute to mTOR activation, and fuel AcCoA synthesis that enters fatty acid synthesis.

When the sensome receptors detect changes in the micromilieu of the brain parenchyma [11] microglia are activated. In LPA-activated microglia this is coupled to an increase in ROS production ([6] and Figure 7) and the exposure of microglia to ROS dictates a need for an antioxidant defense system to limit cell damage. Nrf2 is the master regulator of antioxidant responses and upon translocation to the nucleus Nrf2 induces transcription of target genes that contain the antioxidant response element [79]. Oxidation by ROS or reactive electrophiles of redox sensitive thiols in the inhibitory protein Keap1 allows Nrf2 to accumulate and exert its activity in the nucleus [79]. A second mode of activation that releases Nrf2 from its cytoplasmic anchor Keap1 involves PKC mediated phosphorylation of Nrf2 at Ser^40^. During the present study we have observed an increase of S^40^ phosphorylation of Nrf2 and induction of GCLm expression. GCL is a transcriptional Nrf2 target and an enzyme of central importance for the maintenance of intracellular GSH levels in microglia [80]. In agreement with the induction of GCLm protein expression also GSH levels increased in LPA treated cells (Figure 8), a finding indicative of the induction of a protective pathway to counteract damage of endogenous macromolecules [81].

Our study has also limitations: Experiments were performed in BV-2 cells that show some but not all of the features of primary murine microglia [60]. Some of the effects of LPA on mitochondrial function and GLUT expression were statistically not significant and showed only trends. During lipid and AA analyses we did not perform flux analyses with stable isotope-labeled precursors thus precursor/product relationships remain obscure.

Despite these limitations our findings demonstrate (i) induction of the Akt/mTOR/Hif1α pathway and a concomitant significant increase of lactate secretion from LPA-treated cells, (ii) de-repression of ACC in LPA-treated BV-2 cells and an associated induction of lipogenesis, (iii) higher intracellular concentrations of AAs, and (iv) an LPA-mediated induction of the antioxidant response. The outcome of our experiments identifies the bioactive lipid LPA as a novel mediator of metabolic pathway reprograming in microglia.

## 4. Materials and Methods

### 4.1. Materials

Cell culture medium RPMI1640, fetal calf serum (FCS), antibiotics, and trypsin were from Invitrogen (Waltham, MA, USA). LPA (1-oleoyl-2-hydroxy-sn-glycero-3-phosphate; LPA18:1) was from Sigma-Aldrich (St. Louis, MO, USA). Antibodies were from Cell Signaling Technology (Danvers, MA, USA), Abcam (Cambridge, UK) and Santa Cruz Biotechnology Inc. (Dallas, TX, USA) as listed in Table 1. ROS assay kit was ROS-ID^®^ Total ROS Detection Kit (ENZO Life Sciences, Farmingdale, NY, USA). Glutathione assay kit was GSH-Glo™ Glutathione Assay Kit (Promega Corporation, Madison, WI, USA). Lactate assay kit was EnzyChrom™ Glycolysis Assay Kit (Bioassay Systems, Hayward, CA, USA). Primers were from Qiagen (Hilden, Germany) and Invitrogen (Waltham, MA, USA). Protein estimation kit was Pierce™ BCA Protein Assay Kit (Thermo Fisher Scientific, Waltham, MA, USA). 

### 4.2. BV-2 Microglia

The BV-2 murine microglia cell line was purchased from Banca Biologica e Cell Factory (Genova, Italy). Cells were grown and maintained in RPMI1640 medium supplemented with 10% FCS, 100 units/mL penicillin, and 100 µg/mL streptomycin and 1% L-glutamine (stock 200 mM) and cultured in a humidified incubator under 5% CO_2_ and 95% air. The culture medium was changed to fresh medium every 2–3 days. When cells reached confluency, they were split in new flasks or processed for experiments.

### 4.3. LPA Treatment

**Figure 9:**
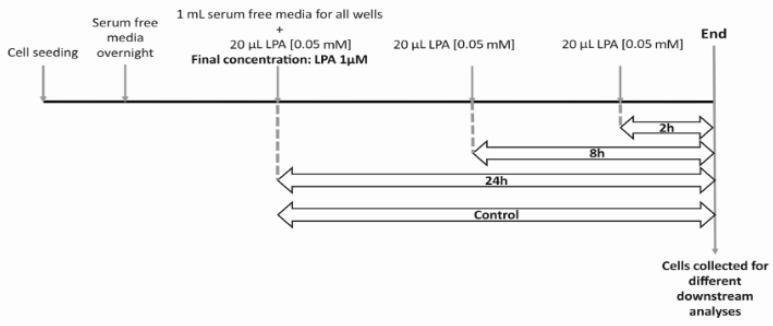
Schematic diagram of the experimental procedure. Cells were seeded and kept serum-free over-night. One mL serum free media was added to all wells and 20 µL LPA [0.05 mM] was added at indicated times for a final concentration of 1 µM. For 5 µM LPA treatment, 1 µL LPA [5 mM] was added to 1 mL media. At the end of the treatment, cells were collected for different downstream analyses.

For LPA treatment, cells were used when they reached approx. 70% confluency. Before treatments, cells were incubated in serum free RPMI medium overnight. The following day, fresh serum free medium was added, and LPA (dissolved in water) was added as shown in Figure 9 below.

### 4.4. Cell Counting

Viability of cells was detected using Guava ViaCount (Merck Millipore, MA, USA). Briefly, the cells were seeded at density of 5 × 10^4^ cells per well in a 12-well plate. Following overnight serum starvation, the cells were treated with LPA for indicated concentration and time periods. At the end of the treatment, cells were washed with PBS, trypsinized, and equal amounts of serum containing medium was added. 50 µL of cell suspension was stained with 150 µL of Guava ViaCount reagent, incubated for 5 min at room temperature and measured using Guava easyCyte 8 Benchtop Flow Cytometer (Merck Millipore). Total viable and dead cells were analyzed.

### 4.5. MTT Assay

The mitochondrial-dependent reduction of MTT to formazan was used to measure cellular metabolic activity. Briefly, the cells were seeded at 1 × 10^4^ cells per well in a 48-well plate. Following overnight serum starvation, the cells were treated with LPA for indicated concentration and time periods. At the end of the treatment, MTT was added to the final concentration of 0.5 mg/mL and incubated for 30 min at 37 °C under standard conditions. Two hundred microliters of lysis buffer (isopropanol/1M HCl (25:1, v/v)) was added with vigorous shaking (1200 rpm, 15 min). 100 µL of it was transferred to 96 well plate. Absorbance was measured at 570 nm on Victor 1420 multilabel counter (Wallac) and corrected for background absorption (650 nm).

### 4.6. Seahorse XF Analyzer Respiratory Assay

Cellular oxygen consumption rate (OCR) was detected using XF Cell Mito Stress Test (Agilent), measured by the extracellular flux analyzer XF96 (Seahorse, Agilent, CA, USA). The sensor cartridge for XF analyzer was hydrated in a 37 °C non-CO_2_ incubator a day before the experiment. Microglia were seeded at a density of 6 × 10^3^ cells per well in Seahorse XFe96 FluxPaks. After overnight serum starvation, cells were treated with the indicated concentrations of LPA for indicated time periods. Prior to OCR measurement, cells were washed and incubated in XF assay medium supplemented with 1 mM sodium pyruvate, 2 mM glutamine and 5 mM D-glucose for 1 h in a 37 °C non-CO_2_ incubator. According to the manufacturer’s instructions, stressors concentrations were optimized and added as follows: 2 μM oligomycin (complex V inhibitor), 0.5 μM carbonyl cyanide-4-(trifluoromethoxy) phenylhydrazone FCCP (uncoupler agent) and 2.5 μM antimycin A (inhibitor of complex I and III). OCR was normalized to protein content (pmoles O_2_/min/µg protein). Experiments were performed in triplicates.

### 4.7. Adenosine Nucleotide and Phosphocreatine Analysis by HPLC

Briefly, the cells were seeded at 1 × 10^5^ per well in a 6-well plate. Following overnight serum starvation, the cells were treated with the indicated concentrations of LPA for indicated time periods. Cells were washed with ice-cold PBS, scrapped off the plates and transferred into Eppendorf tubes. After mild centrifugation the supernatant was discarded and the cells lysed with 200 µL 0.4 M perchloric acid (4 °C). After vortexing and centrifugation at 12000 g 150 µL of the supernatant was neutralized by addition of 15-18 µL of 2 mol/L K_2_CO_3_ at 4 °C. The supernatant obtained after centrifugation was stored at −70 °C until further analysis. Adenosine nucleotides and phosphocreatine (PCr) were measured by HPLC as previously described [82,83]. In brief: Separation was performed with a Hypersil ODS column (5 µm, 250 × 4 mm I.D., equipped with a precolumn; Thermo Electron Corp. Runcorn, Cheshire, UK) using a L-2200 autosampler (injection volume: 40 µL), two L-2130 HTA pumps, and a L-2450 diode array detector (all VWR International, West Chester, PA, USA). Detector signals (absorbance at 214 nm for PCr and 254 nm) were recorded and the program EZchrom Elite (VWR) was used for data acquisition and analysis. Energy charge (EC) was calculated as: EC = [ATP + 1/2ADP]/[ATP + ADP + AMP]. The pellets of the acid extract were resuspended in 0.1 M NaOH for protein determination using the BCA kit (Thermo Scientific, San Jose, CA, USA).

### 4.8. Immunoblotting

For Western blotting experiments, BV-2 cells were seeded onto 6-well plates at a density of 1 × 10^5^ cells per well and serum-starved overnight prior to experiment. Then, cells were treated with the indicated concentrations of LPA for the indicated time periods. Cell lysates were prepared as described in detail in [84] and protein concentrations were determined using BCA kit (Thermo Scientific, San Jose, CA, USA). Fifty micrograms of total protein were separated on 10% SDS-PAGE gels and transferred to polyvinylidene difluoride membranes using electrophoretic transfer (Bio-Rad, Berkeley, CA, USA). Membranes were blocked with 5% *w/v* low-fat milk in TBST and incubated with primary antibodies as indicated. Immunoreactive bands were visualized using corresponding HRP-conjugated secondary antibodies, chemiluminescence HRP substrate development (ECL or ECL plus reagents (Thermo Scientific)) and the Bio-Rad ChemiDoc MP Imaging System (Bio-Rad, Vienna, Austria). In some cases, the membranes were cut, stripped (70 μL β-mercaptoethanol in 10 mL 60 mM Tris/2% SDS buffer, pH 6.8; 55 °C) and re-probed. Anti-β-actin (1:5000) was used as loading control.

### 4.9. RT-qPCR Analysis

Cells were incubated at a density of 1 × 10^5^ cells per well. Following overnight serum starvation, cells were treated with the indicated concentrations of LPA for indicated time periods. Total RNA was extracted using the RNeasy Mini kit (QIAGEN, Hilden, Germany) according to manufacturer’s protocol and quantified using NanoDrop (Thermo Fisher Scientific, Waltham, MA, USA). 1 µg of RNA was reverse-transcribed by using SuperScript^®^ III reverse transcription kit (Invitrogen, Waltham, MA, USA). Quantitative real-time PCR (qPCR) was performed on Applied Biosystems 7900HT Fast Real-Time PCR System using QuantifastTM SYBR^®^ Green PCR kit (QIAGEN, Hilden, Germany. Relative gene expression levels were normalized to hypoxanthineguanine phosphoribosyltransferase (HPRT) and calculated using ΔΔCT method [85]. Primer sequences are listed in Table 1.

### 4.10. Lactate Assay

Lactate content in the supernatant was measured using EnzyChrom™ Glycolysis Assay Kit (ENZO Life Sciences, Switzerland) according to manufacturer’s protocol. Briefly, microglia were seeded in a 12-well plate at a density of 5 × 10^4^ cells per well. Cells were allowed to adhere overnight, then incubated in serum-free medium and treated with LPA (1 or 5 µM as indicated) for indicated time periods. At the end of the treatment, the supernatant was collected and treated with enzyme mix with brief shaking. Optical density was measured at 565nm to quantify lactate content.

### 4.11. Fatty Acid Composition Analysis by Gas Chromatography (GC)

Briefly, the cells were seeded at 1 × 10^5^ per well in a 6-well plate. Following overnight serum starvation, the cells were treated with the indicated concentrations of LPA for the indicated times. At the end of the treatment, the cells were washed with ice cold buffer containing 0.2% fatty acid free bovine serum albumin, sodium chloride (150 mM), Tris (50 mM), adjusted to pH 7.4. Lipids were then extracted twice with hexane/isopropanol (3:1, v/v), dried under a stream of nitrogen and dissolved in 1ml toluene. Lipid subclasses were fractionated by thin layer chromatography (TLC). After addition of the internal standard (pentadecanoic acid), lipids were trans-esterified (1.2 mL toluene and 1 mL boron trifluoride-methanol (20%)) at 110 °C for 1 h. The derivatized lipids were analyzed on Agilent Technologies 5977B gas chromatograph with flame ionization detection (FID) as described previously [86]. Fatty acid concentrations were quantified by peak area comparison with the internal standard and normalized to protein concentration.

### 4.12. Amino Acid Quantification by HPLC

Briefly, the cells were seeded at 1 × 10^5^ per well in a 6-well plate. Following overnight serum starvation, the cells were treated with LPA (1 µM) for indicated time periods. At the end of the treatment, the cells were washed with ice-cold PBS, scrapped off and sonicated in 70 µL H_2_O. 50 µL of 1.5 M HClO_4_ was added and the samples were left aside for 2 min. Then, 1.125 mL water and 25 µL of 2 M K_2_CO_3_ was added. After vortexing and centrifugation (10,000 g, 4 °C, 5 min), the supernatants were collected in glass vials and stored at −70 °C. Following o-phthalaldehyde (OPA) pre-column derivatization, HPLC analysis was performed as described [87]) using a Waters 717 plus Autosampler equipped with a pump delivery system (Shimadzu, LC-20AD) and a scanning fluorescence detector (Waters 474). The amino acid concentrations (calculated by area comparison with external standard curves) were normalized to protein content.

### 4.13. ROS Assay

Intracellular ROS levels were measured using the ROS-ID^®^ Total ROS Detection Kit (ENZO Life Sciences, Switzerland) according to manufacturer’s instructions. In brief, microglia were seeded in black clear bottom 96-well plates at a density of 5 × 10^3^ cells per well. Cells were allowed to adhere overnight, then incubated in serum-free medium and treated with the indicated concentrations of LPA for the indicated times. Thirty minutes before the end of each treatment, the cells were loaded with the ROS detection solution. Fluorescence intensity was measured with excitation and emission wavelengths of 485 nm and 535 nm, respectively.

### 4.14. Glutathione Assay

Intracellular Glutathione content was measured using GSH-Glo™ Glutathione Assay Kit (Promega Corporation, United States) according to the manufacturer’s protocol. Briefly, microglia were seeded in black clear bottom 96-well plates at a density of 5 × 10^3^ cells per well. Cells were allowed to adhere overnight, then incubated in serum-free media and treated with LPA (1 or 5 µM as indicated) for indicated time periods. Then, GSH-Glo™ reagent was added to the plate and incubated for 30 min. After addition of luciferin detection reagent, luminescence was measured to quantify the glutathione content.

### 4.15. Statistical Analysis

Data are expressed as mean ± SEM from at least 3 independent experiments unless specified otherwise. Unpaired Student’s *t*-test (two groups), or one-way ANOVA followed by Bonferroni correction (more than two groups) was used for analysis of statistical significance (using the Graph Pad Prism6 package). All values of *p* < 0.05 were considered significant.

## Figures and Tables

**Table 1:** Antibodies and primers.

**Antibody**	**Company**	**Cat no**	**Dilution**
Phospho Akt (Ser 473)	Cell Signaling Technology	cs-4060	1:1000
Akt	Cell Signaling Technology	cs-9272	1:1000
Phospho Mtor (Ser 2448)	Cell Signaling Technology	cs5536	1:1000
Mtor	Cell Signaling Technology	cs-2972	1:1000
HIF1α	Cell Signaling Technology	cs-14179	1:1000
Phospho AMPK(Thr 172)	Cell Signaling Technology	cs-2535	1:500
AMPK	Cell Signaling Technology	cs-2532	1:1000
Phospho ACC(Ser 79)	Cell Signaling Technology	cs-3661	1:1000
ACC	Cell Signaling Technology	cs-3676	1:1000
Phospho SREBP (Ser 372)	Cell Signaling Technology	cs-9874	1:1000
SREBP1	Abcam	ab44153	1:1000
Phospho NRF2 (Ser 40)	Abcam	ab76026	1:1000
GCLm	Santa Cruz Biotechnology Inc	sc166603	1:1000
β-actin	Santa Cruz Biotechnology Inc	sc47778	1:5000
**Gene**	**Company**	**Cat no**	**Forward/Reverse Primers**
Glut2	Qiagen	QT00103573	
Glut3	Qiagen	QT00159691	
Glut5	Qiagen	QT00148267	
HPRT	Qiagen	QT00166768	
GLUT1	Invitrogen		F: CCGTTCTCCGTCTCGCAG
			R: CTCCCACAGCCAACATGAGG
GLUT4	Invitrogen		F: ATTGTCGGCATGGGTTTCCA
			R: AGCAGGAGGACGGCAAATAG
Hexokinase2	Invitrogen		F: ATCGCCTGCTTATTCACGGAG
			R: TCTGAGAGACGCATGTGGTAG

## Data Availability

Not applicable.

## Supplementary Materials

The following are available online at https://www.mdpi.com/1422-0067/22/4/1968/s1.

## Abbreviations

AA	Amino acid
ACC	Acetyl-CoA carboxylase
AcCoA	Acetyl CoA
ADP	Adenosine diphosphate
AKT	Protein kinase B
Ala	Alanine
AMP	Adenosine monophosphate
AMPK	AMP-activated kinase
ARE	Antioxidant responsive element
Arg	Arginine
Asn	Asparagine
Asp	Aspartic acid
ATP	Adenosine triphosphate
ATX	Autotaxin
Aβ	Amyloid-β
CE	Cholesterylesters
CNS	Central Nervous System
CoA	Coenzyme A
CSF	Cerebrospinal fluid
DAG	Diacylglycerols
ETC	Electron transport chain
FA	Fatty acids
FASN	Fatty Acid Synthase
FCCP	Carbonyl cyanide-4-(trifluoromethoxy) phenylhydrazone
FFA	Free fatty acids
GCLm	Glutamate-cysteine ligase modifier subunit
Gln	Glutamine
GLS	Glutaminase
Glu	Glutamic acid
GLUT	Glucose transporters
Gly	Glycine
GSH	Glutathione
Hif1α	Hypoxia-inducible factor -1α
His	Histidine
HK2	Hexokinase 2
HPLC	High Performance Liquid Chromatography
Ile	Isoleucine
LD	Lipid droplets
Leu	Leucine
LPA	Lysophosphatidic acid
LPAR	Lysophosphatidic acid receptors
LPS	Lipopolysaccharide
Lys	Lysine
mTOR	Mammalian target of rapamycin
Nrf2	Nuclear factor erythroid 2–related factor 2
OCR	Oxygen consumption rate
OPA	o-phthalaldehyde
Orn	Ornithine
PBS	Phosphate Buffered Saline
PCr	Phosphocreatine
PI3K	Phosphoinositide 3-kinases
PL	Phospholipids
Pro	Proline
ROS	Reactive oxygen species
Ser	Serine
SREBP	Sterol regulatory element binding protein
TAG	Triacylglycerols
Tau	Taurine
Thr	Threonine
Trp	Tryptophan
Tyr	Tyrosine
Val	Valine
αKG	Alpha-ketoglutarate
β-Ala	Beta-alanine
