# Supplementary material for: Lysophosphatidic Acid Induces Aerobic Glycolysis, Lipogenesis, and Increased Amino Acid Uptake in BV-2 Microglia
Source: Int J Mol Sci. 2021 Feb 17;22(4):1968. doi: 10.3390/ijms22041968 (PMC7923140; doi:10.3390/ijms22041968)
Supplement: Supplementary file 1 [file ijms-22-01968-s001.pdf]

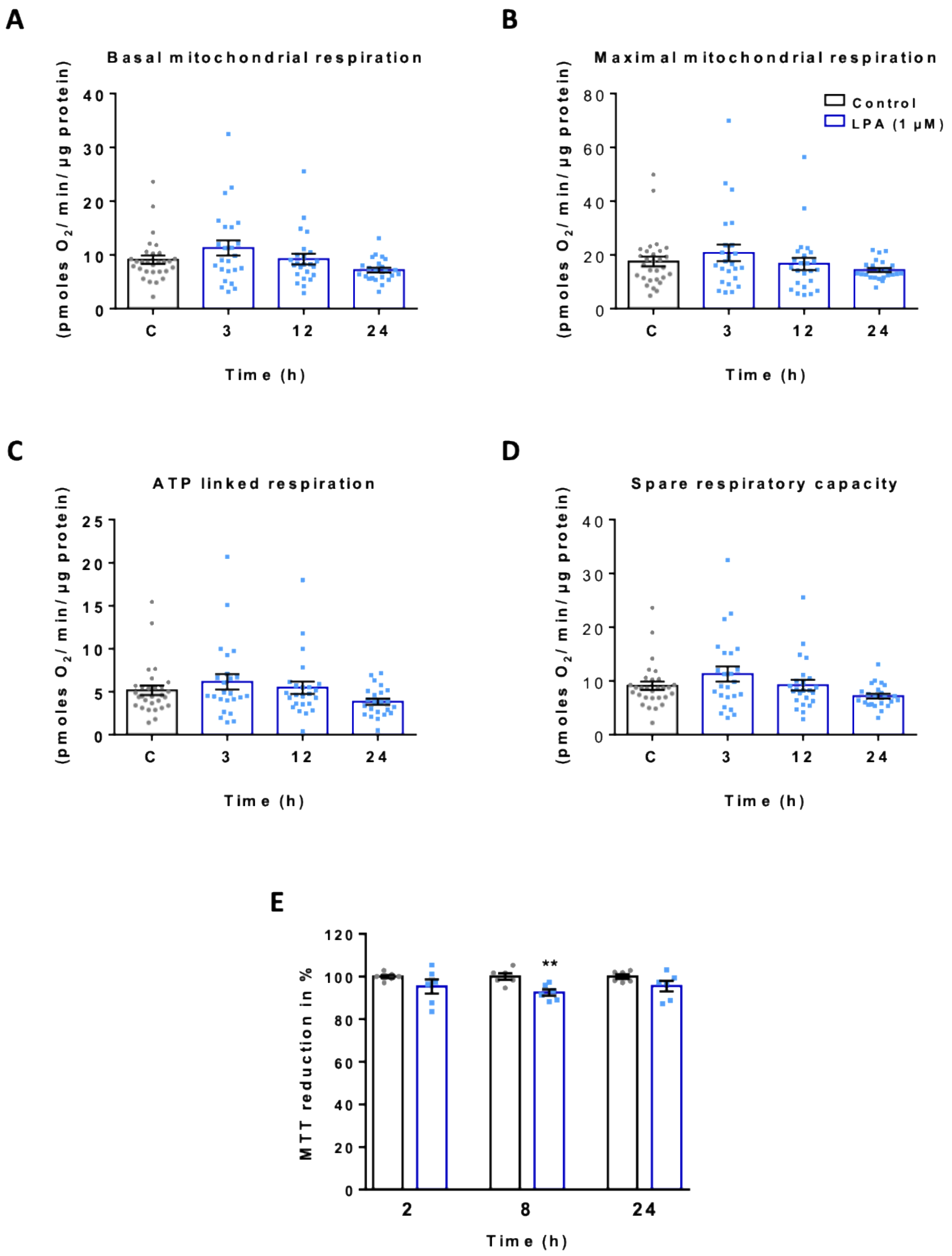

Figure S1. Effect of LPA (1 µM) on mitochondrial respiration and MTT reduction of BV-2 microglia. Oxygen consumption rate (OCR) in the absence and presence of 1 µM LPA for the indicated times was detected using the XF Cell Mito Stress Test to assess fundamental parameters of mitochondrial function. Bar graphs show (A) basal mitochondrial respiration, (B) maximal mitochondrial respiration, (C) ATP linked respiration, and (D) spare respiratory capacity. (E) Effect of LPA (1 µM) on MTT reduction in cells was compared to control. Results are shown as mean values ± SEM of 3 independent experiments; one-way ANOVA with Bonferroni correction.

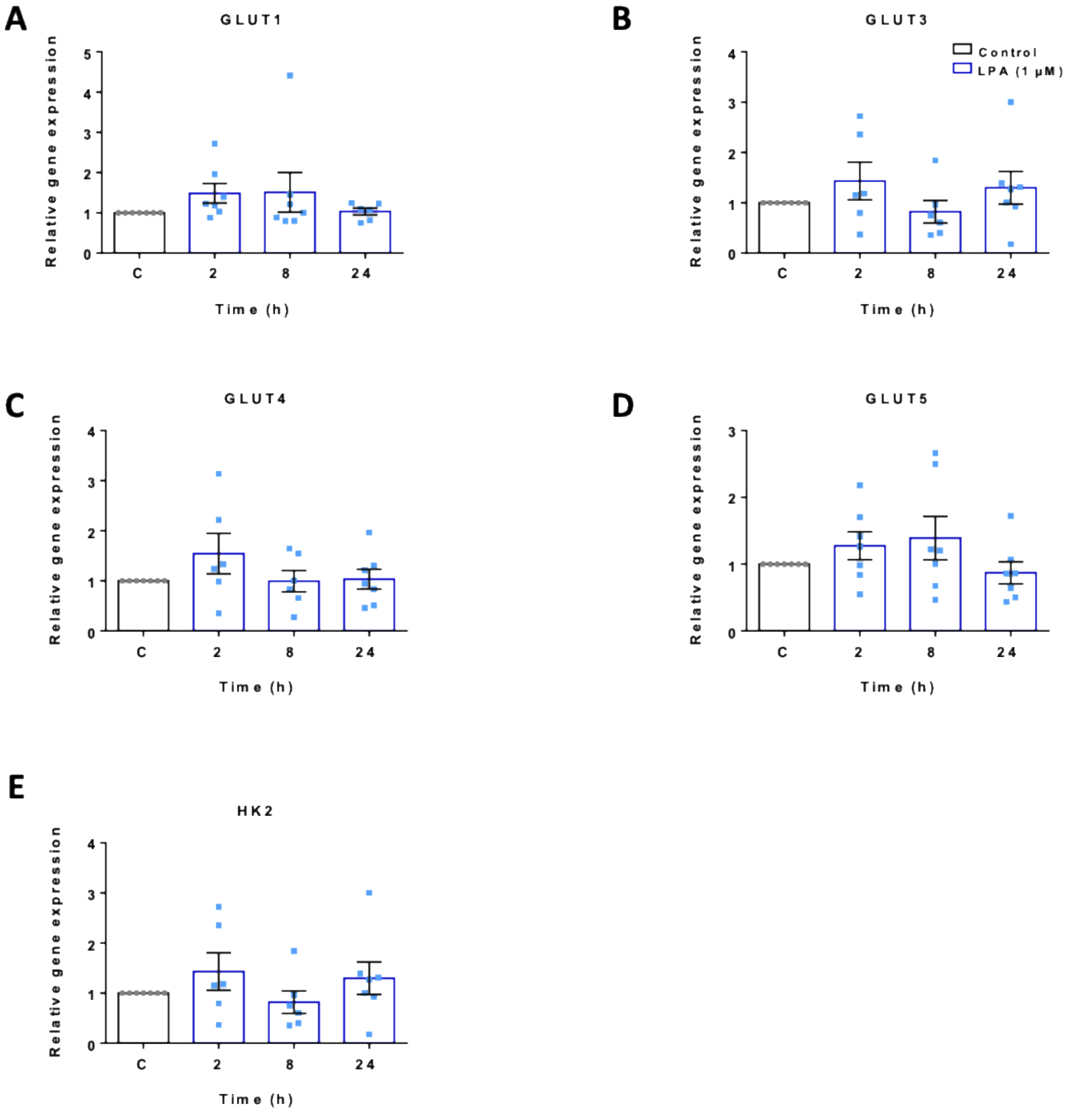

Figure S2: Gene expression of glucose transporters and hexokinase 2 in LPA (1 µM) -treated BV-2 cells. Serum-starved BV-2 cells were treated with 1 µM LPA for the indicated times and gene expression of glucose transporters (A) GLUT1, (B) GLUT3, (C) GLUT4, and (D) GLUT5, and (E) hexokinase 2 was evaluated by qPCR analysis. Hypoxanthine-guanine phosphoribosyltransferase (HPRT) was used as housekeeping gene. Expression was calculated using the 2^{-ddCt} method. Results are presented as mean values ± SEM of 3 independent experiments (one-way ANOVA with Bonferroni correction).

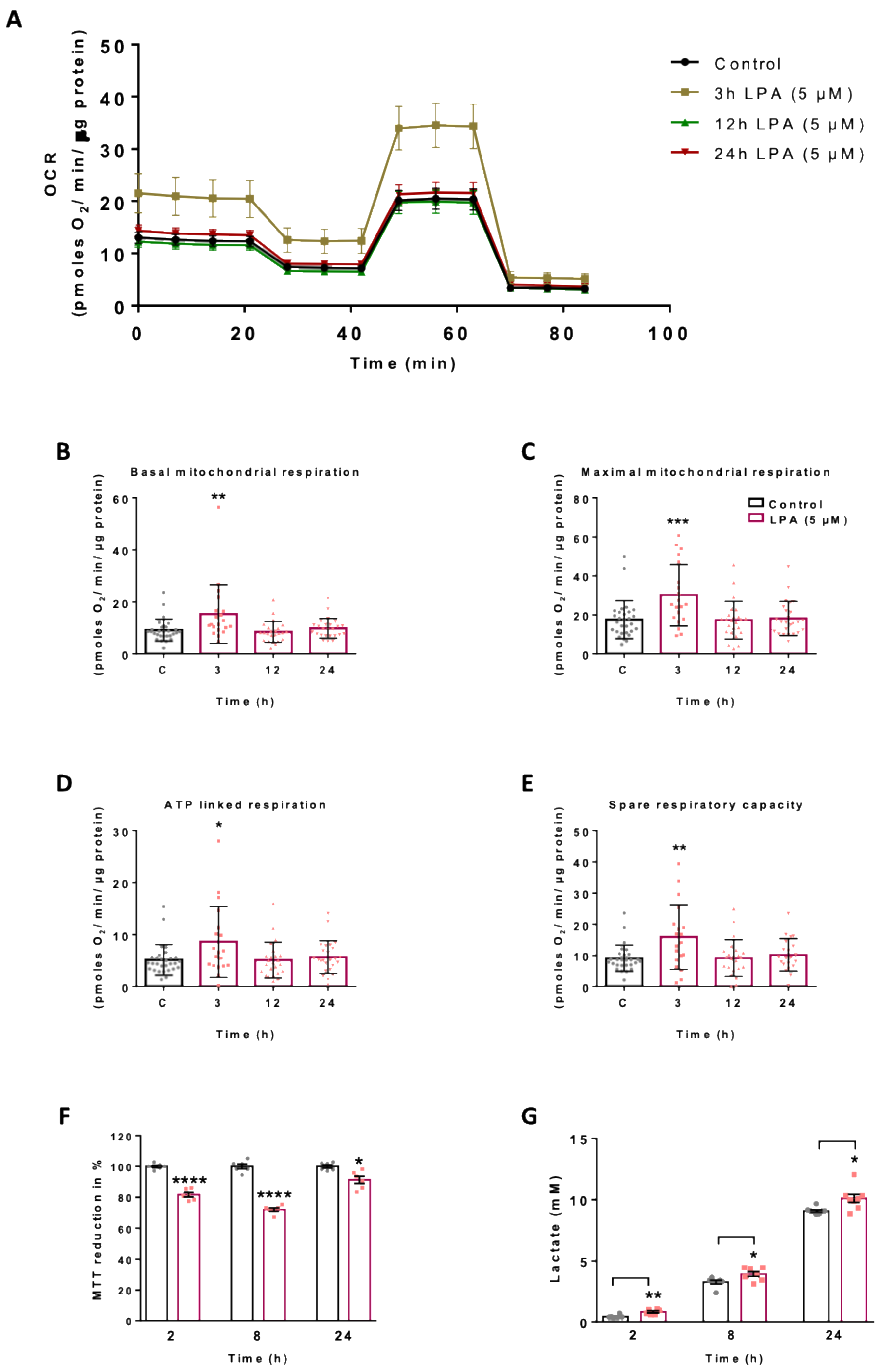

Figure S3: Effect of LPA (5 µM) on mitochondrial respiration, MTT reduction, and lactate secretion of BV-2 microglia. (A) Oxygen consumption rate (OCR) in the absence and presence of 5 µM LPA for the indicated times was detected using the XF Cell Mito Stress Test. Cells were treated with 2 µM oligomycin, 0.5 µM FCCP, and 2.5 µM antimycin A in XF assay medium to assess fundamental parameters of mitochondrial function. Bar graphs show (B) basal mitochondrial respiration, (C) ATP linked respiration, (D) maximal mitochondrial respiration, and (E) spare respiratory capacity. (F) Effect of LPA (5 µM) on MTT reduction in cells was compared to control. (G) Lactate content of BV-2 cells treated with 5 µM LPA was measured by EnzyChrom™ Glycolysis Assay Kit and compared to their appropriate controls. Bar graph represents mean values ± SEM of 3 independent experiments; (*p<0.05, **p<0.01, *** p<0.001, ****p<0.0001 compared to control; one-way ANOVA with Bonferroni correction).

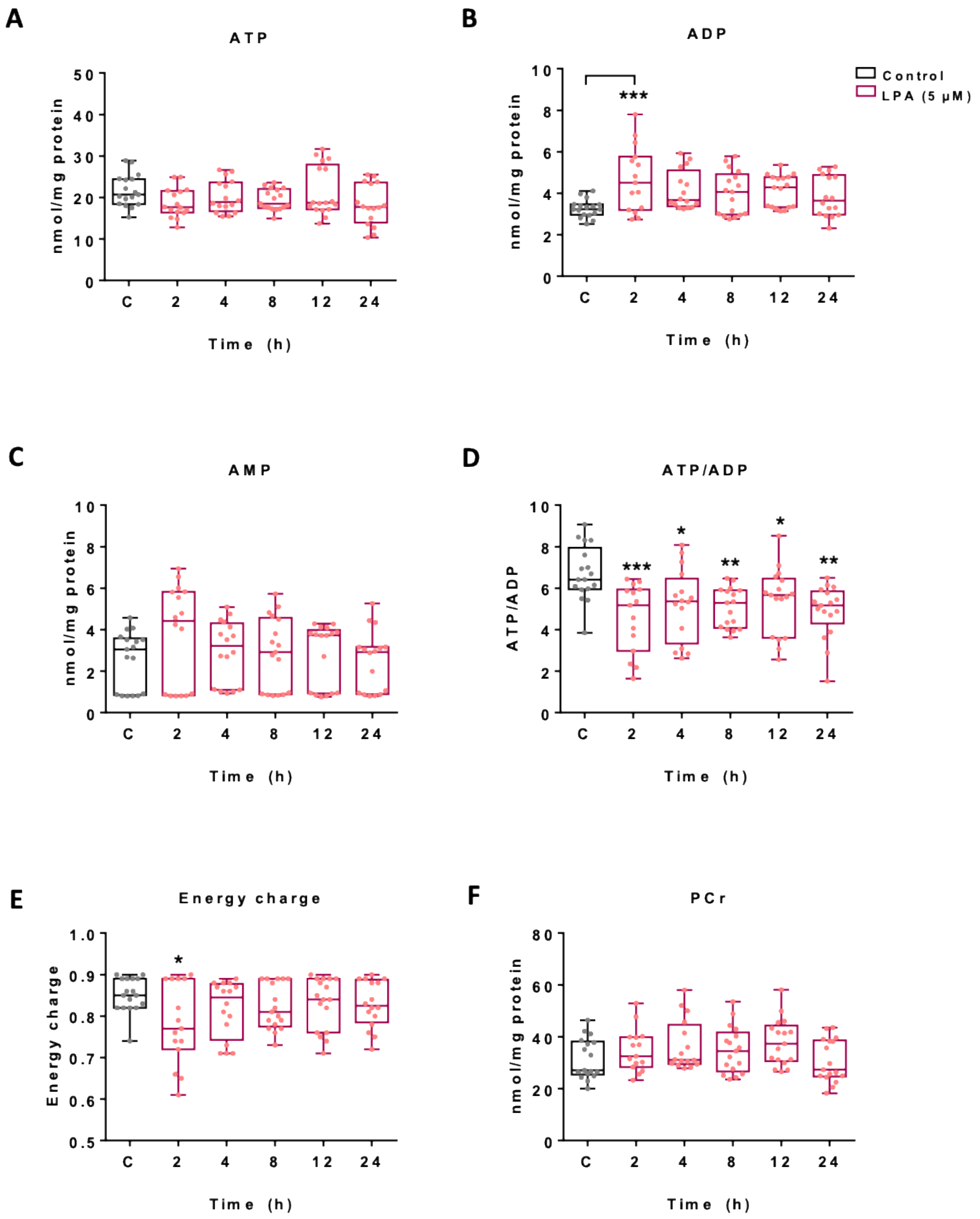

Figure S4: Effect of LPA (5 μM) on adenine nucleotide and phosphocreatine content in BV-2 microglia. Total cellular adenine nucleotide and phosphocreatine (PCr) content of BV-2 microglia in response to LPA (5 μM) treatment was quantified using HPLC. Graphs represent the concentration of (A) ATP, (B) ADP, (C) AMP, (D) ATP/ADP ratio, (E) the energy charge (EC) calculated as EC = ([ATP+1/2ADP]/[ATP+ADP+AMP]) and (F) phosphocreatine concentration. Results are presented as mean values ± SEM of 3 independent experiments. (*$p<0.05$, **$p<0.01$, ***$p<0.001$, compared to control; one-way ANOVA with Bonferroni correction).

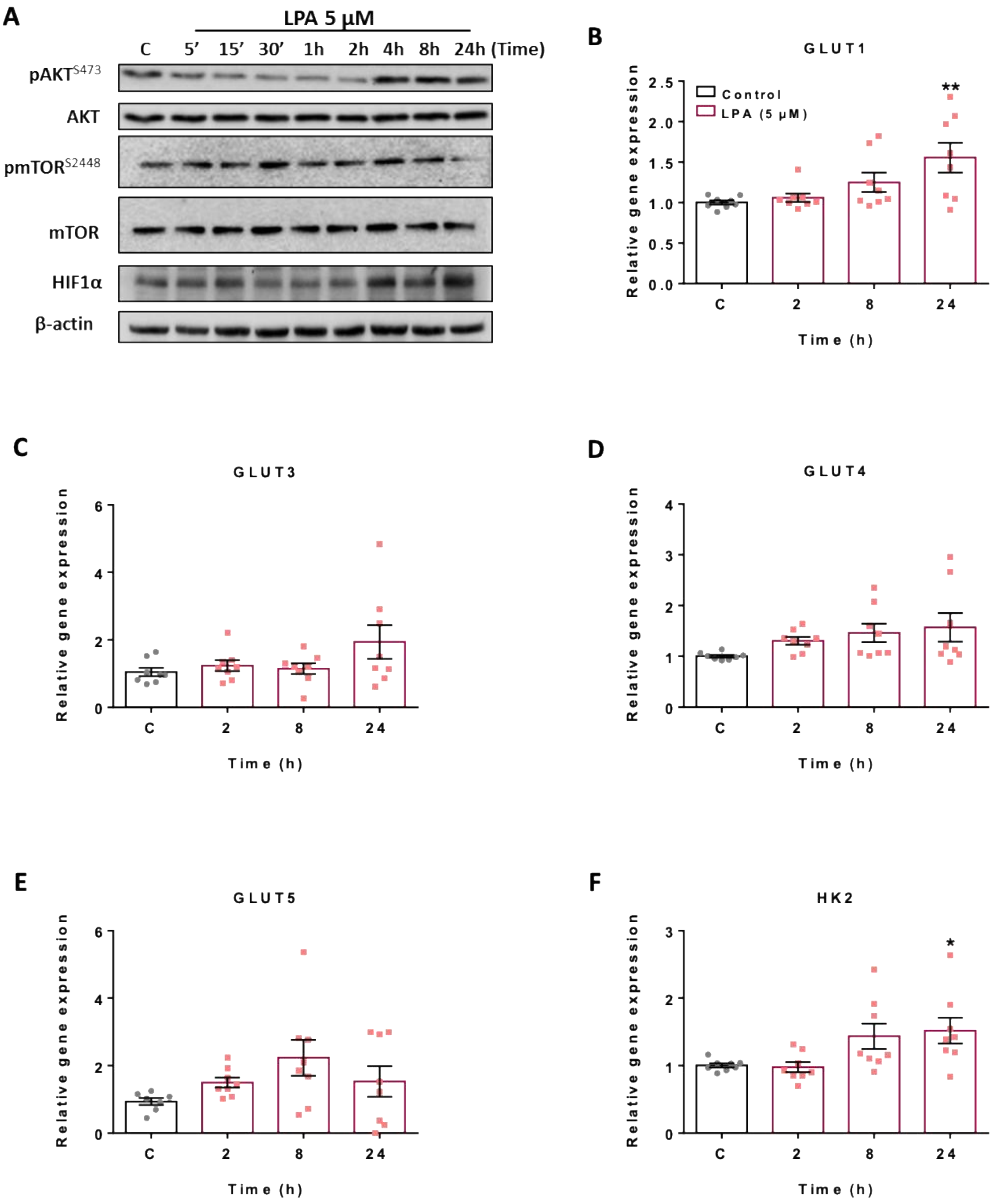

Figure S5: Immunoblot analysis of proteins involved in glycolysis and gene expression of glucose transporters and hexokinase upon treatment with LPA (5 µM). Serum-starved BV-2 cells were incubated in the absence (c) and presence of 5 µM LPA for the indicated time points. Expression levels of (A) phospho AKT (pAKT), phospho mTOR (pmTOR), and HIF1α relative to β-actin were examined by immunoblotting. (B) Serum-starved BV-2 cells were treated with 5 µM LPA for the indicated times and gene expression of glucose transporters (B) GLUT1, (B) GLUT3, (C) GLUT4, and (D) GLUT5, and (E) hexokinase 2 was evaluated by qPCR analysis. Hypoxanthine-guanine phosphoribosyltransferase (HPRT) was used as housekeeping gene. Expression was calculated using the 2^{-ddCt} method. Data are show as mean ± SEM; of 3 independent experiments. (*p<0.05, **p<0.01 compared to control; one-way ANOVA with Bonferroni correction).

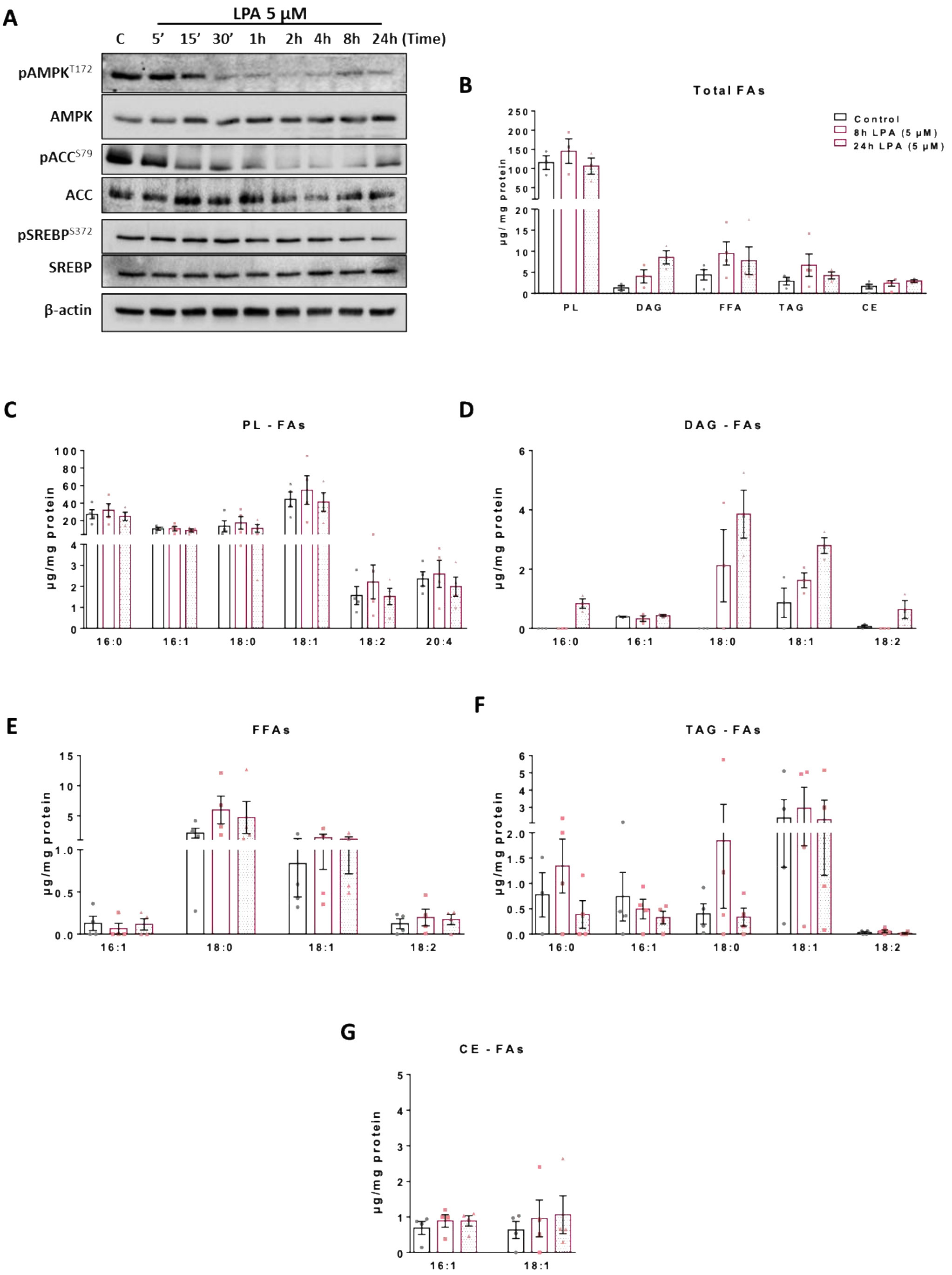

Figure S6: Phosphorylation status of AMPK, ACC, and SREBP and quantitation of fatty acid species in lipid subclasses in LPA (5 µM) - treated BV-2 cells. BV-2 cells were serum-starved overnight and incubated in the absence ('c') and presence of 5 µM LPA for the indicated times. Expression levels of (A) phospho AMPK (pAMPK), phospho ACC (pACC), and phospho SREBP (pSREBP) relative to β-actin are shown. (B) Quantitation of total FAs present in the indicated lipid species (PL, phospholipids; DAG, diacylglycerols; FFA, free fatty acids; TAG, triacylglycerols; CE, cholesterylesters). Individual FA content in each lipid class: (C) phospholipids, (D) diacylglycerols, (E) free fatty acids, (F) triacylglycerols, and (G) cholesterylesters. Bar graph represent mean ± SEM of four independent experiments; one-way ANOVA with Bonferroni correction).

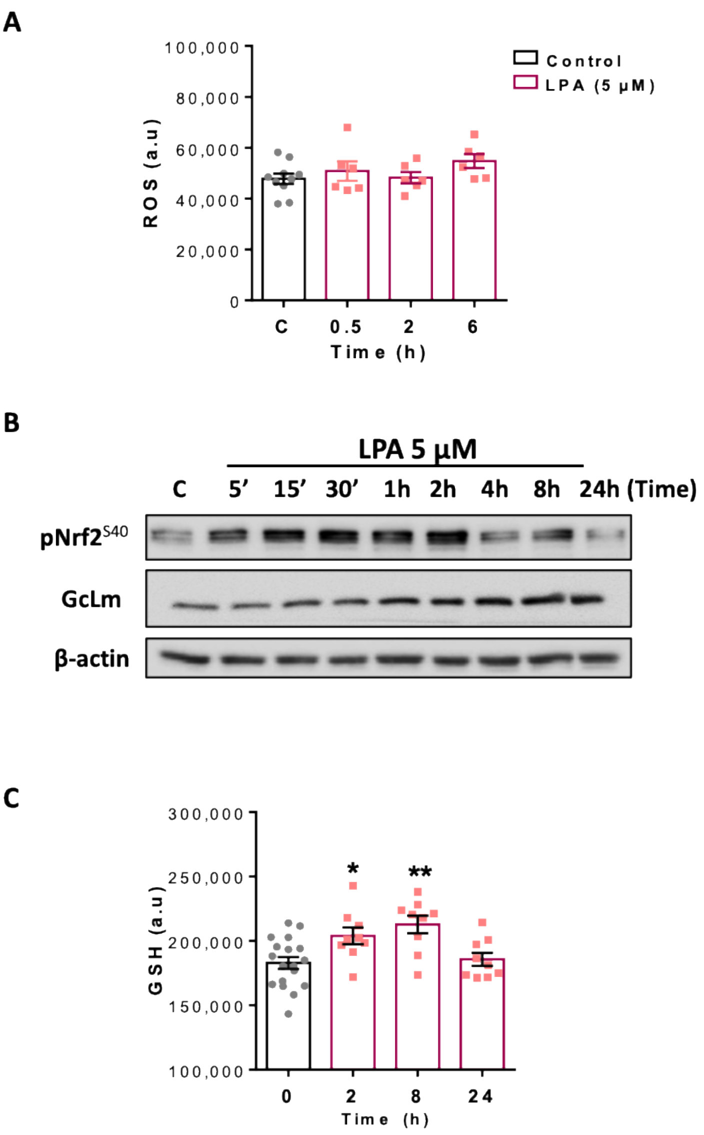

Figure S7: Characterization of the antioxidant response in LPA (5 µM) - treated BV-2 cells. (A) Reactive oxygen species (ROS) of control and LPA (5 µM) treated cells was quantified using the ROS-ID® Total ROS Detection kit (a.u. arbitrary units). (B) Immunoblot analysis of phosphoNrf2 (Ser40) and GCLm upon treatment with LPA. β-actin was used as loading control. (C) Glutathione (GSH) concentration of LPA (5 µM) treated cells was quantified with GSH-Glo Assay kit and compared with control. Results are presented as mean values ± SEM of three independent experiments. (*p<0.05, **p<0.01, compared to control; one-way ANOVA with Bonferroni correction).
